# Characterization of a Bacteriocin-like Substance from *Loigolactobacillus coryniformis* WYQ-2 and Its Antibacterial Activity Against *Escherichia coli*

**DOI:** 10.3390/vetsci13090909

**Published:** 2026-09-04

**Authors:** Yuqi Wang, Binglun Sui, Boran Zhang, Xinyue Wang, Lianjun Fu, Wenlong Dong, Baishuang Yin, Man Yan, Wanli Sha

**Affiliations:** 1 College of Animal Science and Technology, Jilin Agricultural Science and Technology College, Jilin 132101, China; wangyuqi@jlnku.edu.cn (Y.W.); suibinglun@jlnku.edu.cn (B.S.); zhangboran@jlnku.edu.cn (B.Z.); wangxinyue@jlnku.edu.cn (X.W.); fulianjun@jlnku.edu.cn (L.F.); dongwenlong888@jlnku.edu.cn (W.D.); yinbaishuang@jlnku.edu.cn (B.Y.); 2 Jilin Provincial Key Laboratory of Preventive Veterinary Medicine, Jilin Agricultural Science and Technology College, Jilin 132101, China; 3 Jilin Province Technology Innovation Center of Pig Ecological Breeding and Disease Prevention and Control, Jilin Agricultural Science and Technology College, Jilin 132101, China; 4 Jilin Province Cross Regional Cooperation Technology Innovation Center of Porcine Main Disease Prevention and Control, Jilin 132101, China

**Keywords:** *Escherichia coli*, *Loigolactobacillus coryniformis*, bacteriocin-like, antimicrobial mechanism

## Abstract

*Escherichia coli* is a common porcine diarrheal pathogen transmitted via the fecal–oral route through contaminated environments and feed. This study evaluated the antimicrobial potential of a bacteriocin-like substance produced by *Loigolactobacillus coryniformis* WYQ-2 isolated from pickled vegetables. Its physicochemical stability, molecular mass, and antimicrobial spectrum were characterized, and the mode of action against *Escherichia coli* was investigated through intracellular ATP quantification, live/dead staining, and scanning electron microscopy (SEM). The substance exhibited broad-spectrum antibacterial activity, with the strongest inhibition against *Escherichia coli* (the diameter of the inhibition zone reached 41.35 mm). It remained stable under prolonged UV irradiation (0–2.5 h), under heating at 100 °C, and within the pH range of 3.0–9.0. Molecular mass determination revealed a low-molecular-weight peptide (<2.7 kDa). Protease susceptibility tests showed activity was reduced by trypsin and papain, slightly sensitive to proteinase K, yet relatively stable toward other tested proteases. Mechanistically, ATP levels declined markedly within 0.5–2 h of treatment, while live/dead staining and SEM confirmed membrane disruption, deformation, and surface indentations in treated *Escherichia coli* cells. These findings demonstrate that the WYQ-2-derived bacteriocin-like substance possesses favorable physicochemical traits and exerts antibacterial action primarily by compromising membrane integrity, highlighting its promise as a natural alternative for controlling porcine diarrheal diseases.

## Simple Summary

The escalating antibiotic resistance crisis necessitates the urgent development of safe and effective alternatives for the prevention and control of swine-origin bacterial diarrhea. In the present study, *Loigolactobacillus coryniformis* WYQ-2, isolated from pickled vegetables, was employed to systematically characterize the physicochemical properties and antimicrobial activity of its bacteriocin-like WYQ-2cin. The substance displayed potent antibacterial activity against Gram-negative pathogens, including diarrheagenic *Escherichia coli*, primarily through disruption of cell membrane integrity, and retained stability under high temperature, acidic/alkaline conditions, and UV irradiation. These findings indicate that BLIS WYQ-2cin possesses potential as a natural antibiotic alternative for the prevention and control of porcine bacterial diarrhea.

## 1. Introduction

*Escherichia coli* (*E. coli*) is characterized as a bacillus, a Gram-negative bacterium within the family Enterobacteriaceae [1]. It ranks among the most highly adaptable and pathogenically diverse bacterial species, associated with a spectrum of human infections that include gastrointestinal and extraintestinal diseases [2]. Additionally, *E. coli* constitutes a component of the commensal intestinal microbiota in humans and other mammals [3]. However, certain pathotypes of *E*. *coli*, particularly enterotoxigenic *E. coli* (ETEC), are recognized as causative agents of acute diarrhea and mortality in swine herds, with suckling and recently weaned piglets being the most susceptible [4,5], typically presenting with profuse watery diarrhea, severe dehydration, and metabolic acidosis [6]. Transmission is mediated through contaminated water sources, feedstuffs, pen surfaces, and transport vehicles [7], which complicates the complete eradication of outbreaks once they become established within farm environments. Critically, the heavy reliance on antimicrobials to mitigate clinical episodes inadvertently accelerates the selection and dissemination of drug-resistant clones, thereby inflicting considerable financial losses on the production sector. Traditionally, antibiotics have been the primary tool to meet this need; however, their widespread use has paradoxically driven the very resistance that now threatens their efficacy. Accordingly, its robust transmission potential underscores the urgent need for effective control measures.

Antibiotics serve as a cornerstone in the fight against infectious diseases and have significantly contributed to global health. However, their excessive use has directly fueled the spread of antimicrobial resistance (AMR), posing a growing public health crisis [8]. Therefore, identifying strategies to reduce reliance on antibiotics and develop adjunctive treatments has become particularly critical. Lactic acid bacteria (LAB)—a group of Gram-positive microorganisms naturally present in fermented foods and employed as probiotics [9]—demonstrate considerable potential in inhibiting pathogens and are generally recognized as safe (GRAS) regardless of their isolation source [10]. Historically, they have been widely applied in food processing [11]. *Loigolactobacillus coryniformis* (*L. coryniformis*), a member of LAB, exerts its antimicrobial activity largely through the production of bacteriocin substances. Bacteriocins are a class of ribosomally synthesized antimicrobial proteins or peptides [12]. Their growth-inhibitory effect against specific microbial communities becomes particularly pronounced at effective concentrations. According to their molecular architecture, heat tolerance, and the presence or absence of post-translational modifications, bacteriocins are generally grouped into four main categories: Class I comprises lantibiotics that contain modified amino acid residues after translation; Class II consists of small peptides that are heat-stable and unmodified; Class III includes larger proteins that are heat-labile; and Class IV covers cyclic peptides that remain stable under heat [13]. Thus, research on the antimicrobial substances produced by *L*. *coryniformis* provides a promising direction for developing novel and safe antibacterial strategies.

The inhibitory effect of bacteriocin substances produced by lactic acid bacteria (LAB), against the common pathogenic foodborne bacterium *E. coli* has been extensively and thoroughly studied by scholars around the world, specifically in the research context of industrial and academic food processing safety technology development. In this study, bacteriocin-like substances derived from *L*. *coryniformis* isolated from pickled vegetables were investigated. Their antimicrobial activity and stability under various conditions were evaluated through multiple assays, including molecular weight determination and analysis of physicochemical properties, demonstrating significant antibacterial efficacy against *E. coli.* However, existing research has primarily focused on their potential applications in food, with limited attention paid to their antibacterial mechanisms against *E. coli* strains responsible for porcine diarrhea in livestock farming. Therefore, considerable potential remains for further exploration into the underlying antimicrobial mechanisms. It should be emphasized that bacteriocins previously documented from *L. coryniformis* generally fall within a molecular mass range of 3–10 kDa and display inhibitory activity that is largely directed against Gram-positive species. In marked contrast, the bacteriocin identified in the present work not only exerts potent activity against Gram-negative bacteria but also possessing a low molecular weight. These unique characteristics strongly highlight the innovative aspects of this study. Furthermore, most existing studies have been confined to preliminary observations of antibacterial activity, whereas the present investigation comprehensively evaluated the feasibility of this bacteriocin as an antibiotic substitute for the prevention and control of porcine diarrhea through whole-genome sequencing, time-kill kinetics assays, and physicochemical characterization, thereby opening new avenues for its use in veterinary settings.

## 2. Materials and Methods

### 2.1. Isolation and Characterization of Bacteria

A total of 15 pickled vegetable samples were collected from Liaoning, China, and each sample was homogenized in sterile physiological saline. After serial dilution, the homogenates were spread onto Man–Rogosa–Sharpe (Qingdao, Haibo Technology Co., Ltd., China) agar plates and incubated at 37 °C for 48 h. Based on differences in colony morphology, 22 single colonies were picked and individually inoculated into MRS broth, followed by incubation at 37 °C with shaking for 24 h to allow fermentation. After centrifugation of the fermentation broth, the supernatant was collected, and the antimicrobial activity of all isolates was preliminarily screened by the agar well diffusion method using *E. coli* ATCC 25922 as the indicator strain. The strain exhibiting the largest inhibition zone was designated WYQ-2. In MRS broth until turbidity developed. Under aseptic conditions, the culture was inoculated onto MRS agar plates and incubated statically at 37 °C for 24 h. Individual colonies were isolated to obtain pure single colonies. Following repeated purification via the streak plate method, preliminary identification was performed using Gram staining. Under oil immersion microscopy, the colonies were observed as purple-blue rods, confirming that WYQ-2 is a Gram-positive bacterium. The cell suspension was combined with glycerol (20%) and subsequently preserved at −80 °C. For strain identification, DNA was extracted and amplified by PCR with universal primers targeting the 16S rRNA gene [14]. The resulting amplicons were sent to General Biotechnology Co., Ltd. (Anhui, China) for 16S rRNA gene sequencing. The obtained sequence was subjected to BLAST (http://www.ncbi.nlm.nih.gov/BLAST/ (accessed on 10 June 2026)) analysis against existing sequences in the GenBank database, with the accession number PZ840592.

### 2.2. Preparation and Assessment of Bacteriocin Antibacterial Activity

To prepare the cell-free supernatant (CFS), *L. coryniformis* WYQ-2 was grown in 300 mL of MRS broth at 37 °C for 48 h under continuous shaking. After centrifugation of the culture at 3500× *g* for 15 min using a TFL16M centrifuge (Yancheng Kaite Experimental Instrument Co., Ltd., Yancheng, China), the supernatant was collected and subsequently filtered through a 0.22-μm polyethersulfone membrane. This filtrate was then concentrated to one-tenth of its original volume using a rotary evaporator set at 55 °C and 50 rpm. To rule out any acid-derived inhibitory effects, the concentrate was brought to pH 6.0 with NaOH. Catalase was then introduced at a final level of 1 mg/mL, and the resulting mixture was held at 37 °C for 4 h to remove any possible interference from hydrogen peroxide.

*E. coli* ATCC 25922 was cultured in LB medium at 37 °C with agitation to the logarithmic stage, and the cell density was then brought to 1 × 10^8^ CFU/mL. Antibacterial activity was determined using the agar well diffusion technique. Briefly, 50 μL of this indicator culture was applied evenly across the surface of Mueller–Hinton (MH) agar plates. Using sterile Oxford cups, wells measuring 8 mm in diameter were created, and 200 μL of the concentrated CFS was dispensed into each well. The plates were then allowed to equilibrate at room temperature for 2 h to enable pre-diffusion, after which they were incubated at 37 °C for 12 h. The diameters of the resulting inhibition halos were subsequently recorded using a vernier caliper.

### 2.3. Purification and Molecular Weight Determination of Bacteriocin

*L. coryniformis* WYQ-2 was added to 1500 mL of MRS broth at a 2% (*v*/*v*) inoculum level and incubated at 37 °C for 48 h. The resulting CFS was prepared as detailed in Section 2.2. This CFS was then concentrated by a factor of 10 via rotary evaporation at 55 °C, followed by dialysis at 4 °C for 24 h against deionized water using a dialysis membrane with a 3.5 kDa molecular-weight cutoff. After dialysis, both the retentate and the permeate were independently concentrated, and their antibacterial activity was assessed by the agar diffusion method, with *E. coli* ATCC 25922 serving as the indicator strain.

To further determine the molecular mass of the active component, Tricine-SDS-PAGE was carried out. A discontinuous gel was prepared, comprising a 4% stacking gel, a 10% spacer gel, and an 18% separating gel, following the protocol described in [15]. As the reference, a low-molecular-mass peptide marker spanning 2.7–40 kDa (Sangon Biotech, Shanghai, China) was used. The electrophoresis run was performed in a water bath cooled with dry ice. Voltage was applied in stages: initially at 30 V after loading 15 μL of sample and 10 μL of marker, until the marker migrated into the spacer gel; then raised to 80 V until the dye front entered the separating gel; and finally set to 120 V until the run finished. After electrophoresis, the gel was immersed for 30 min in a fixative composed of 50% methanol, 10% acetic acid, and 40% distilled water, rinsed three times with distilled water, and then subjected to staining with Coomassie Brilliant Blue R-250 for 1–2 h. Destaining was finally carried out using a solution of 20% ethanol, 10% glacial acetic acid, and 70% distilled water, which was applied until the protein bands became clearly visible. To detect antibacterial activity, an identical gel was run in parallel under the same conditions. After the run, this gel was washed with distilled water, then laid on a fresh plate and covered with MH agar that had been inoculated with *E. coli* ATCC 25922. After incubation at 37 °C for 12 h, the formation of inhibition zones was examined.

### 2.4. Antibacterial Spectrum of WYQ-2

The Oxford cup method was employed to evaluate the antimicrobial activity of the bacteriocin-like substance produced by strain WYQ-2 against eight different bacterial strains. The tested strains comprised six Gram-negative bacteria—*E. coli* ATCC 25922, *E. coli* 324C3, *E. coli* Ef-a, *E. coli* G-18, *Klebsiella pneumoniae TY-6* and *Salmonella typhimurium* ATCC 14028—as well as two Gram-positive bacteria—*Staphylococcus aureus* ATCC 25923 and *Enterococcus faecalis* ATCC 29212. The test strains were cultured in LB broth (*E. coli* ATCC 25922, *E. coli* 324C3, *E. coli* Ef-a, *E. coli* G-18, *S. aureus* ATCC 25923, *S. Typhimurium* ATCC 14028, *K. pneumoniae* TY-6) or the BHI broth (*E. faecalis* ATCC 29212) at 37 °C to obtain bacterial suspensions with a density of 1 × 10^8^ CFU/mL. Following this, the prepared cell suspensions of individual strains were uniformly applied over the surface of MH agar plates. After the Oxford cup procedure was completed, the plates were kept at 37 °C for 16 h, and the clear zone diameters around each well were subsequently determined with a vernier caliper. For mechanistic studies, *E. coli* ATCC 25922 (purchased from the American Type Culture Collection, ATCC, Manassas, VA, USA) was used as the model indicator strain. The wild-type strains used for antimicrobial spectrum testing—*E. coli* 324C3, Ef-a, and G-18—were all clinical strains isolated and preserved by our laboratory from fecal samples of diarrheal piglets (14–35 days of age) on large-scale pig farms in Jilin Province.

### 2.5. Physicochemical Properties and Stability of the WYQ-2-Derived Bacteriocin

#### 2.5.1. Protease Stability Assay

Five aliquots of bacteriocin-like substance were subjected to protease treatment to exclude interference from proteolytic enzymes, with an enzyme-free setup as the control. Papain, trypsin, pepsin, proteinase K, and pronase were separately used to treat bacteriocin. Trypsin is a serine protease that specifically cleaves peptide bonds at the carboxyl terminus of lysine (Lys) and arginine (Arg) residues [16]. Papain is a cysteine protease that preferentially recognizes and hydrolyzes peptide bonds involving amino acids with bulky hydrophobic side chains (e.g., phenylalanine and leucine) as well as basic residues (arginine and lysine) [17]. Proteinase K is a broad-spectrum serine protease that predominantly cleaves peptide bonds at the carboxyl side of aliphatic and aromatic hydrophobic amino acids [18]. Pepsin is an aspartic protease exhibiting optimal activity under acidic conditions (pH 1.0–3.0), preferentially hydrolyzing peptide bonds adjacent to aromatic amino acids (phenylalanine, tyrosine, and tryptophan) and hydrophobic residues [19]. Pronase is a nonspecific protease mixture produced by Streptomyces griseus, containing multiple endopeptidase and exopeptidase activities, and is capable of extensively degrading proteins into free amino acids [20]. Each reaction system was brought to its respective optimal pH with 1 mol/L NaOH or HCl. The mixtures were then incubated at 37 °C for 2 h with the enzyme added to a final level of 1 mg/mL, and the enzymatic reaction was subsequently terminated by heating at 90–100 °C for 10 min. This step aimed to eliminate antibacterial activity originating from endogenous enzyme-like substances produced by BLIS WYQ-2cin, thereby confirming that the antimicrobial activity was primarily derived from bacteriocin-like substance. Finally, the residual antibacterial activity was determined using *E*. *coli* ATCC 25922 as the indicator strain.

#### 2.5.2. Thermal Stability

Six aliquots of bacteriocin-like substance were incubated at different temperatures (–20 °C, 4 °C, 37 °C, 60 °C, 80 °C, and 100 °C) for 1 h, with the sample kept at 37 °C serving as the control. After treatment, all samples were equilibrated to room temperature, and their antibacterial activity was assessed against *E. coli* ATCC 25922.

#### 2.5.3. pH Stability

The pH of six separate aliquots of the bacteriocin-like substance was brought to values spanning 4.0–9.0 by adding 1 mol/L NaOH or HCl, and the aliquots were then incubated in a water bath at 37 °C for 2 h. The original sample (pH ≈ 3.0) was used as the control. After incubation, the pH of each sample was readjusted to the initial value (pH ≈ 3.0). Antibacterial activity was then tested against *E. coli* ATCC 25922.

#### 2.5.4. UV Light Stability

For exposure periods of 0, 0.5, 1, 1.5, 2, and 2.5 h, five aliquots of the bacteriocin-like preparation were exposed to 254-nm UV light. An unexposed sample served as the control. After UV irradiation, the antibacterial activity of each aliquot was determined using *E. coli* ATCC 25922 as the indicator strain.

### 2.6. Growth Curve of WYQ-2

To monitor the growth behavior and define the different growth phases of *L. coryniformis* WYQ-2, the strain was cultured in MRS broth with a 2% (*v*/*v*) inoculation level and shaken at 37 °C. Over a total period of 36 h, culture samples were withdrawn every 2 h. The optical density at 600 nm (OD_600_) and the pH variation in the culture were recorded with a spectrophotometer. At the same time, the antibacterial activity in the culture supernatant was assessed against the indicator strain *E. coli* ATCC 25922 using the agar well diffusion technique.

### 2.7. Time-Kill Curve of WYQ-2

*E. coli* ATCC 25922 was grown in 40 mL of LB broth with a 100 μL inoculum at 37 °C until reaching the exponential phase, after which the cell density was adjusted to 1 × 10^8^ CFU/mL. The bacterial culture was then centrifuged (8000× *g*, 5 min), the supernatant removed, and the resulting pellet washed three times with sterile saline. In parallel, *L. coryniformis* WYQ-2 was cultured in 40 mL of MRS broth with a 100 μL inoculum at 37 °C to the logarithmic stage, followed by centrifugation at 8000× *g* for 5 min and collection of the supernatant. A 20 mL portion of the obtained supernatant was combined with 20 mL of the washed *E. coli* cells, and the mixture was incubated for 24 h. At 2 h intervals, aliquots were withdrawn and the OD_600_ was recorded with a spectrophotometer. As a control, MRS broth was mixed with the indicator bacterium and subjected to the same procedures. Each experiment was conducted in triplicate, and the average values were calculated.

### 2.8. Whole-Genome Sequencing and Bioinformatic Analysis of WYQ-2

The genomic DNA of *L*. *coryniformis* WYQ-2 was subjected to whole-genome sequencing. The strain was first activated by shaking cultivation in MRS broth at 37 °C for 24 h, followed by centrifugation, and the resulting pellet was submitted to Shanghai Major Bio-Pharm Technology Co., Ltd., Shanghai, China for sequencing. To comprehensively characterize its genome, a hybrid sequencing strategy was employed using both the Oxford Nanopore PromethION platform (for long reads) and the Illumina NovaSeq6000 platform (for short reads). A hybrid assembly was conducted using Unicycler v0.4.8 to combine long and short reads, after which the draft genome was refined with Pilon v1.22 via short-read alignment. Additionally, a separate long-read-only assembly was carried out with Canu v1.5 and subsequently refined through iterative polishing with Racon v3.4.3. Following assembly, gene prediction for protein-coding sequences (CDSs) was carried out with Prodigal v2.6.3, whereas tRNA and rRNA genes were detected using tRNAscan-SE v2.0 and Barrnap v0.9, respectively. The identification of biosynthetic gene clusters involved in secondary metabolism was performed with BAGEL4, and potential specific functional genes were screened using GenBlastA v1.0.4. Finally, a circular genomic map illustrating the key genomic features was generated with Circos v0.69-6. The raw sequencing data are available under the NCBI accession number JBYEQQ000000000.

### 2.9. Quantification of Intracellular ATP Levels

To assess cytoplasmic membrane permeability, the intracellular ATP concentration of *E*. *coli* ATCC 25922 was measured following the protocol of Rao et al. [21]. *E. coli* ATCC 25922 cells were cultivated to the logarithmic phase, harvested by centrifugation (8000× *g*, 5 min, 4 °C), and after removal of the supernatant, washed thrice with PBS and resuspended. Equal volumes of the bacterial suspension and WYQ-2 were co-incubated for 0.5, 1, and 2 h. The cells were then collected by centrifugation, after which the supernatant was removed, and the pellet was rinsed three times with PBS prior to a further centrifugation step for recovery. The resulting cell pellet was resuspended in 1 mL of extraction solution and disrupted by ultrasonication in an ice-water bath using an ultrasonic cell disruptor set at 200 W, with a pulse cycle of 2 s on and 1 s off, for a total sonication duration of 1 min. A sample treated identically with PBS served as the control. After disruption, the lysate was centrifuged at 8000× *g* for 10 min at 4 °C, and the supernatant was transferred, mixed thoroughly with chloroform by vigorous shaking, and centrifuged again at 8000× *g* for 3 min at 4 °C. The resulting supernatant was collected for analysis. Finally, ATP content was quantified in accordance with the manufacturer’s instructions (Solarbio, Beijing, China).

### 2.10. Live/Dead Cell Assay of BLIS WYQ-2cin

The SYTO-9/PI dual-staining approach serves as an effective means to reveal membrane integrity loss in *E. coli* resulting from bacteriocin exposure. The principle of this method relies on the differential response of the two fluorescent dyes to membrane integrity: SYTO-9 permeates intact cell membranes, binds to nucleic acids, and emits green fluorescence, thereby labeling all bacterial cells; whereas propidium iodide (PI) enters only cells with compromised membrane structures, producing red fluorescence that specifically indicates dead or severely membrane-damaged cells. The staining procedure was carried out as specified by the protocol of the SYTO-9/PI Bacterial Viability Kit (Servicebio, Wuhan, China). *E. coli* ATCC 25922 was grown to the exponential phase (3–4 h), pelleted by centrifugation at 7378× *g* for 5 min, and rinsed three times with sterile 0.9% saline prior to re-suspension. The cell suspension was then combined with the bacteriocin-like preparation and incubated at 37 °C for 2 h, with a parallel sample lacking the bacteriocin-like substance serving as the negative reference. After incubation, the cell pellets were rinsed an additional three times with 0.9% saline and subsequently incubated in the dark with SYTO-9 and propidium iodide (PI) for 15 min. Finally, the stained cells were examined under a fluorescence microscope with dark-field optics, where viable cells appeared green and membrane-compromised cells displayed red fluorescence.

### 2.11. Scanning Electron Microscope (SEM)

To explore the morphological changes in *E. coli* ATCC 25922 upon exposure to BLIS WYQ-2cin, exponential-phase cells (OD_600_ ≈ 0.5) were harvested via centrifugation (7378× *g*, 5 min), washed three times with PBS, and then re-suspended. The bacterial suspension was combined with an equal volume of the bacteriocin-like preparation and incubated at 37 °C for 2 h, whereas the control group received PBS only. Following incubation, the cells were harvested again by centrifugation. The pellet was then incubated overnight at 4 °C in 2.5% glutaraldehyde for fixation, followed by gradient dehydration in a graded ethanol series (30%, 50%, 70%, 80%, 90%, and 100%) at 4 °C, with each step lasting 15 min. After drying, the specimens were mounted onto metal stubs, coated with a conductive gold layer by sputter-deposition, and finally viewed under an SEM to assess surface structural changes.

### 2.12. Statistical Analysis

All experiments were conducted in triplicate to ensure the reliability of the obtained data. Results are expressed as the mean ± standard deviation. Statistical analyses were performed using SPSS 27.0 software. One-way ANOVA was applied first, and if a significant difference was detected (*p* < 0.05), Duncan’s multiple range test was then applied for post hoc comparisons. In the figures, significant differences are denoted by distinct lowercase letters (a, b, c…). Preliminary data processing and descriptive statistics were carried out using Microsoft Excel.

## 3. Results

### 3.1. Phylogenetic Tree

**Figure 1:**
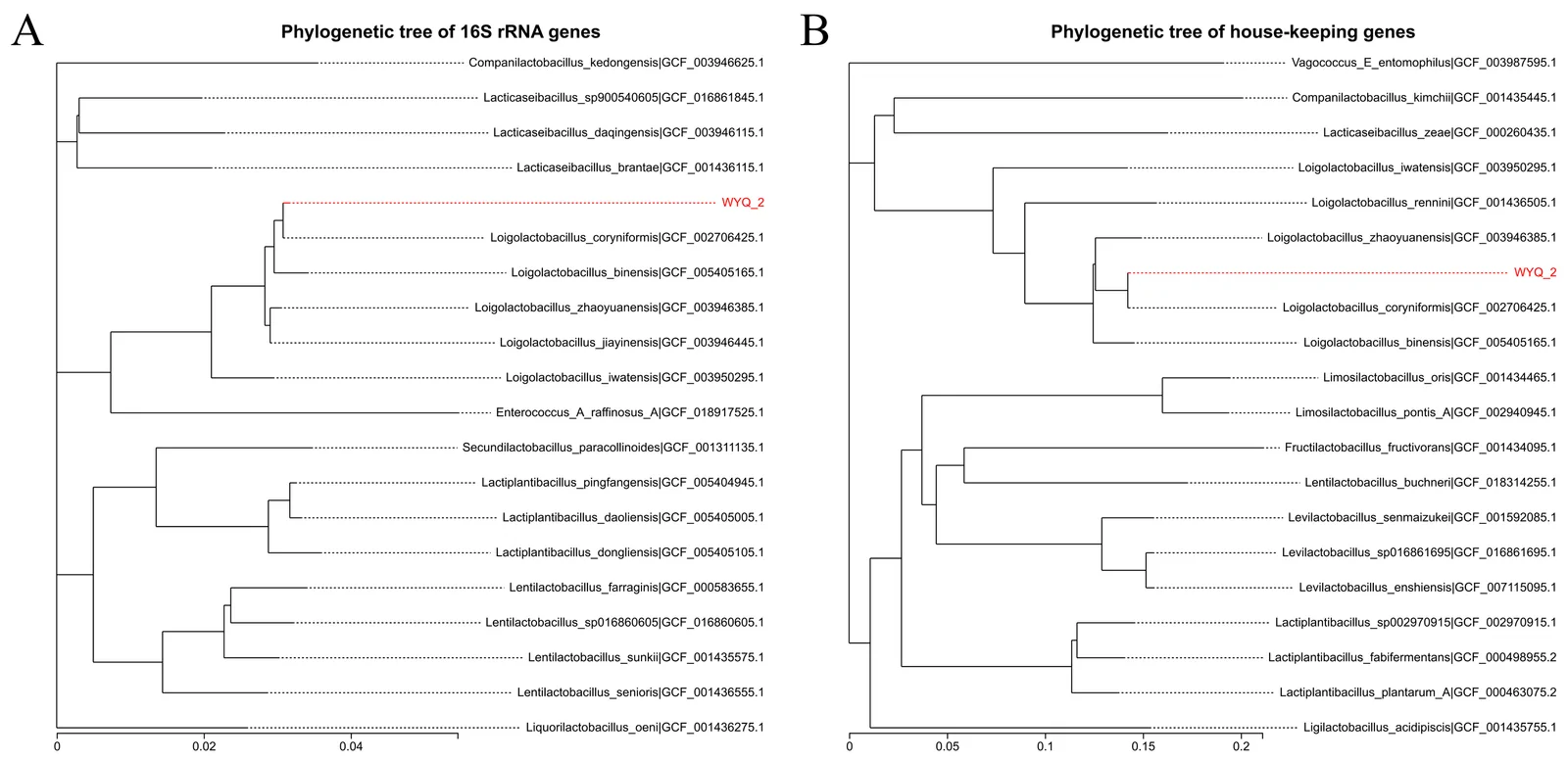
(**A**) displays the phylogenetic tree for WYQ-2 constructed from the 16S rRNA gene, and (**B**) displays the phylogenetic tree constructed from housekeeping genes.

According to the sequencing results from Anhui General Biotechnology Co., Ltd., the 16S rRNA gene sequence of strain WYQ-2 exhibited 99% identity to that of *L*. *coryniformis*. Phylogenetic trees constructed from the 16S rRNA gene (Figure 1A) and housekeeping genes (Figure 1B) both revealed that WYQ-2 reliably clustered within the same clade as reference strains of *L. coryniformis*. These results demonstrate that strain WYQ-2 can be identified as *L*. *coryniformis*.

### 3.2. Antimicrobial Identification and Spectrum Analysis

Prior to conducting antibacterial assays, we employed catalase treatment to exclude hydrogen peroxide-mediated activity and adjusted the pH to neutralize organic acid interference. The CFS retained significant antimicrobial activity, indicating that the inhibitory effect was attributable neither to hydrogen peroxide nor organic acids. Antimicrobial spectrum testing (Table 1) revealed that BLIS WYQ-2cin exhibits broad-spectrum inhibitory activity, with markedly stronger activity against Gram-negative bacteria than against Gram-positive species. Among the tested strains, the bacteriocin demonstrated excellent specificity and high potency against *E. coli* ATCC 25922. These findings confirm its strong inhibitory effectiveness against *E. coli* (the inhibition zone against *E. coli* ATCC 25922 reached 41.35 mm, and those against the tested Gram-negative bacteria ranged from 34.70 to 41.35 mm) and highlight its potential value for applications in both the food and livestock industries.

**Table 1:** The antimicrobial spectrum of BLIS WYQ-2cin.

Indicator Strains	Source	Gram	Inhibition Zone (mm)
**Gram-negative bacteria**			
*E. coli*	ATCC 25922	−ve	41.35 ± 0.54
*E. coli*	324C3	−ve	40.58 ± 0.71
*E. coli*	Ef-a	−ve	39.17 ± 0.70
*E. coli*	G-18	−ve	40.27 ± 0.62
*K. pneumoniae*	TY-6	−ve	36.82 ± 0.42
*S. typhimurium*	ATCC 14028	−ve	34.70 ± 0.52
**Gram-positive bacteria**			
*E. faecalis*	ATCC 29212	+ve	29.21 ± 0.69
*S. aureus*	ATCC 25923	+ve	31.33 ± 0.43

Note: Antibacterial activity was determined using the Oxford cup method, with 200 μL of a 10-fold concentrated cell-free supernatant (×10) added to each well. The diameter of the inhibition zone included the outer diameter of the Oxford cup (8 mm). Data are expressed as mean ± standard deviation. The negative control (blank MRS broth concentrated 10-fold) showed no zone of inhibition. Strain sources: ATCC strains were purchased from the American Type Culture Collection; clinical isolates were isolated and preserved by our laboratory from fecal samples of diarrheic piglets collected at large-scale pig farms in Jilin Province.

### 3.3. Effects of Protease, Temperature, pH, and UV Irradiation on Antimicrobial Activity

**Figure 2:**
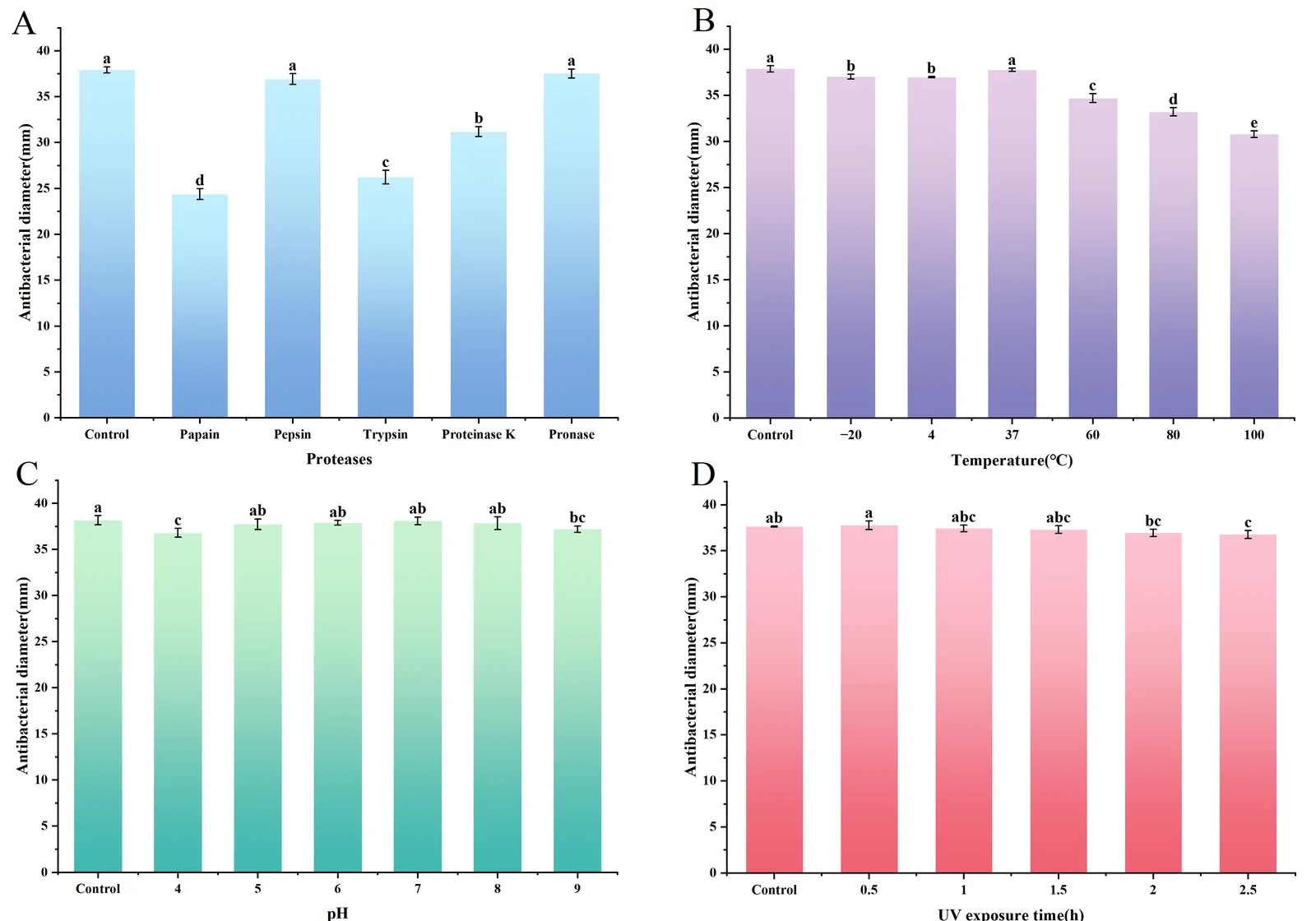
Antimicrobial activity of BLIS WYQ−2cin under various protease treatments, temperatures, pH values, and UV exposure. (**A**) Effect of various protease treatments on the antibacterial activity of BLIS WYQ−2cin; (**B**) Effect of temperature on the antibacterial activity of BLIS WYQ−2cin; (**C**) Effect of pH on the antibacterial activity of BLIS WYQ−2cin; (**D**) Effect of UV exposure time on the antibacterial activity of BLIS WYQ−2cin. Data are presented as the mean of three replicates, and error bars indicate the standard deviation. Different letters indicate statistically significant differences (*p* < 0.05); the same letters indicate no significant difference (*p* ≥ 0.05).

Figure 2 demonstrates the stability of the BLIS WYQ-2cin produced by *L*. *coryniformis* WYQ-2 under various environmental conditions. Protease sensitivity analysis (Figure 2A) revealed that BLIS WYQ-2cin is highly susceptible to papain and trypsin, as indicated by a significant reduction in the inhibition zone diameter from 37.58 mm (control) to 23.74 mm (papain) and 25.41 mm (trypsin), respectively. In contrast, the bacteriocin-like substance displayed limited susceptibility to proteinase K, whereas it exhibited markedly greater resistance to pepsin and pronase, accompanied by only a marginal reduction in antibacterial activity. Thermal stability tests (Figure 2B) showed that BLIS WYQ-2cin maintains stable antibacterial activity across a broad temperature range from −20 °C to 100 °C, confirming its pronounced thermostability. The pH stability profile (Figure 2C) indicated that BLIS WYQ-2cin retains antimicrobial activity within the pH range of 3.0–9.0 (control pH ≈ 3.0). The bacteriocin-like substance remained largely stable across a broad pH range, exhibiting negligible fluctuation in activity. These results demonstrate that the bacteriocin-like substance retains potent activity across various conditions (100 °C, pH 3.0–9.0, 2.5 h UV irradiation), substantiating its capacity to endure the environmental stresses prevalent in the gastrointestinal tract and fermented food matrices. Furthermore, after continuous exposure to 254 nm ultraviolet irradiation for up to 2.5 h, the antibacterial activity of BLIS WYQ-2cin remained stable, with no significant change in inhibition zone diameter compared to the unexposed control (Figure 2D), demonstrating its notable photostability and tolerance. In summary, BLIS WYQ-2cin possesses a combination of favourable activity in various milieus, excellent photostability, outstanding thermostability, and selective sensitivity to specific proteases.

### 3.4. Growth Curve of L. coryniformis WYQ-2 and Inhibition Curve of E. coli ATCC 25922 Under BLIS WYQ-2cin Treatment

**Figure 3:**
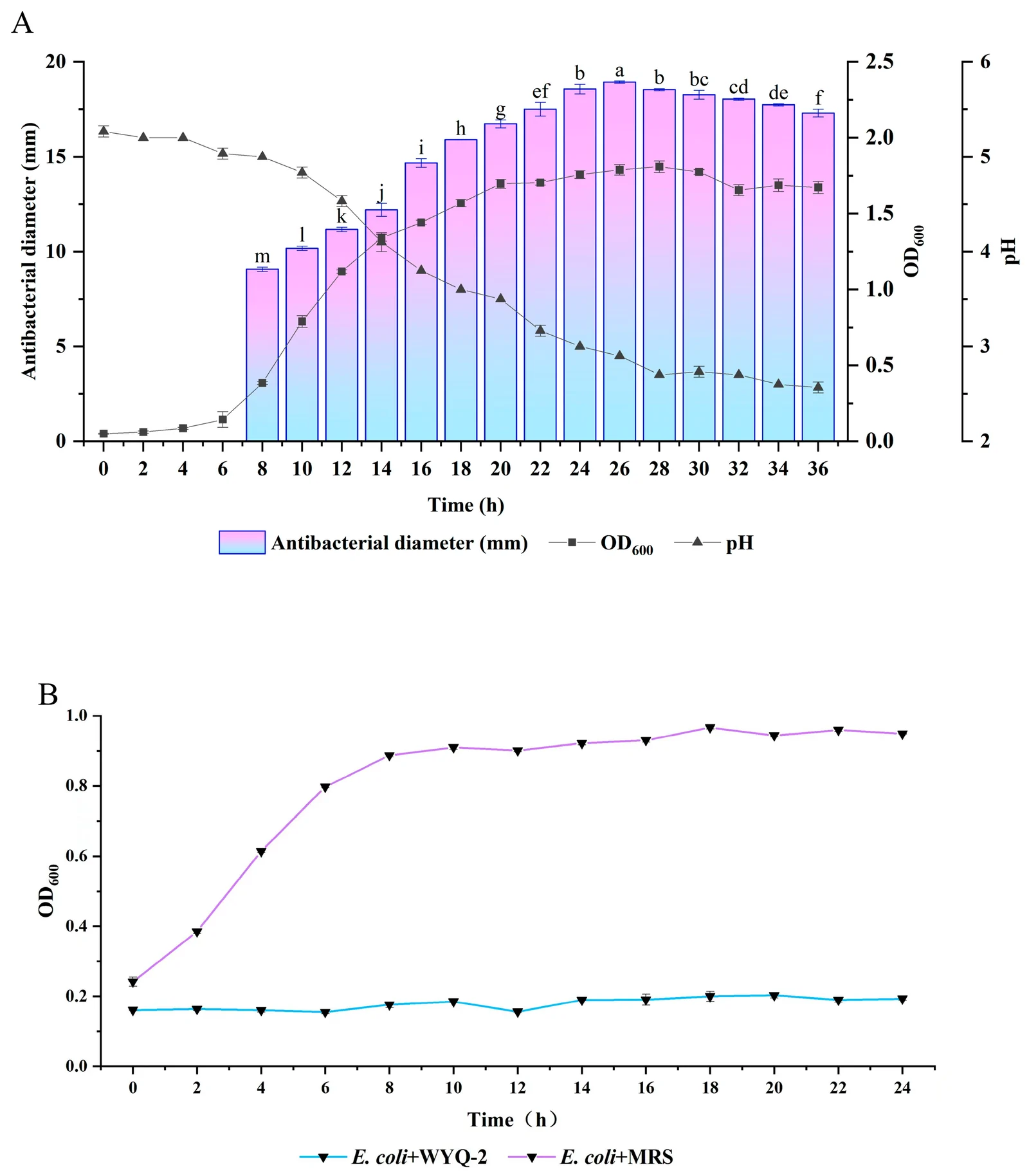
Growth curve and inhibition curve. (**A**) Growth curve of WYQ-2 over time. (In the figure, the histogram represents the diameter of the inhibition zone, the square line chart represents OD600, and the triangle line chart represents pH value.) (**B**) Inhibitory effect of BLIS WYQ-2cin on the growth of *E. coli* ATCC 25922. Different letters indicate statistically significant differences (*p* < 0.05); the same letters indicate no significant difference (*p* ≥ 0.05).

During a 36 h monitoring period, growth kinetics of *L*. *coryniformis* WYQ-2 and *E*. *coli* ATCC 25922 were analyzed at a total of 19 time points (including 0 h). Based on OD600 measurements (Figure 3A), the growth of WYQ-2 entered a plateau phase around 20 h, indicating a transition from the logarithmic growth phase to a stable stage. The acid-production capability of the strain was reflected by a significant drop in pH at 8 h, which eventually stabilized at approximately 2.5. Antibacterial activity analysis revealed that the antimicrobial substance produced by WYQ-2 was first detected at 8 h (inhibition zone diameter: 9 mm) and reached its peak activity at 26 h (antibacterial diameter: 18.9 mm). These results indicated that BLIS WYQ-2cin accumulated predominantly during the stationary phase and reached its maximal yield, which is consistent with the characteristic biosynthetic pattern of bacteriocins as secondary metabolites. To clarify the inhibitory effect of BLIS WYQ-2cin on the target bacterium, the dynamic change in OD600 of *E. coli* ATCC 25922 was monitored in the system. The results showed that the addition of BLIS WYQ-2cin significantly suppressed the growth of this pathogen, leading to a flattened growth curve (Figure 3B). These findings indicate that BLIS WYQ-2cin effectively interferes with the normal growth progression of *E. coli* ATCC 25922.

### 3.5. Genomic Characteristics and Coding Sequence Analysis of L. coryniformis WYQ-2

**Figure 4:**
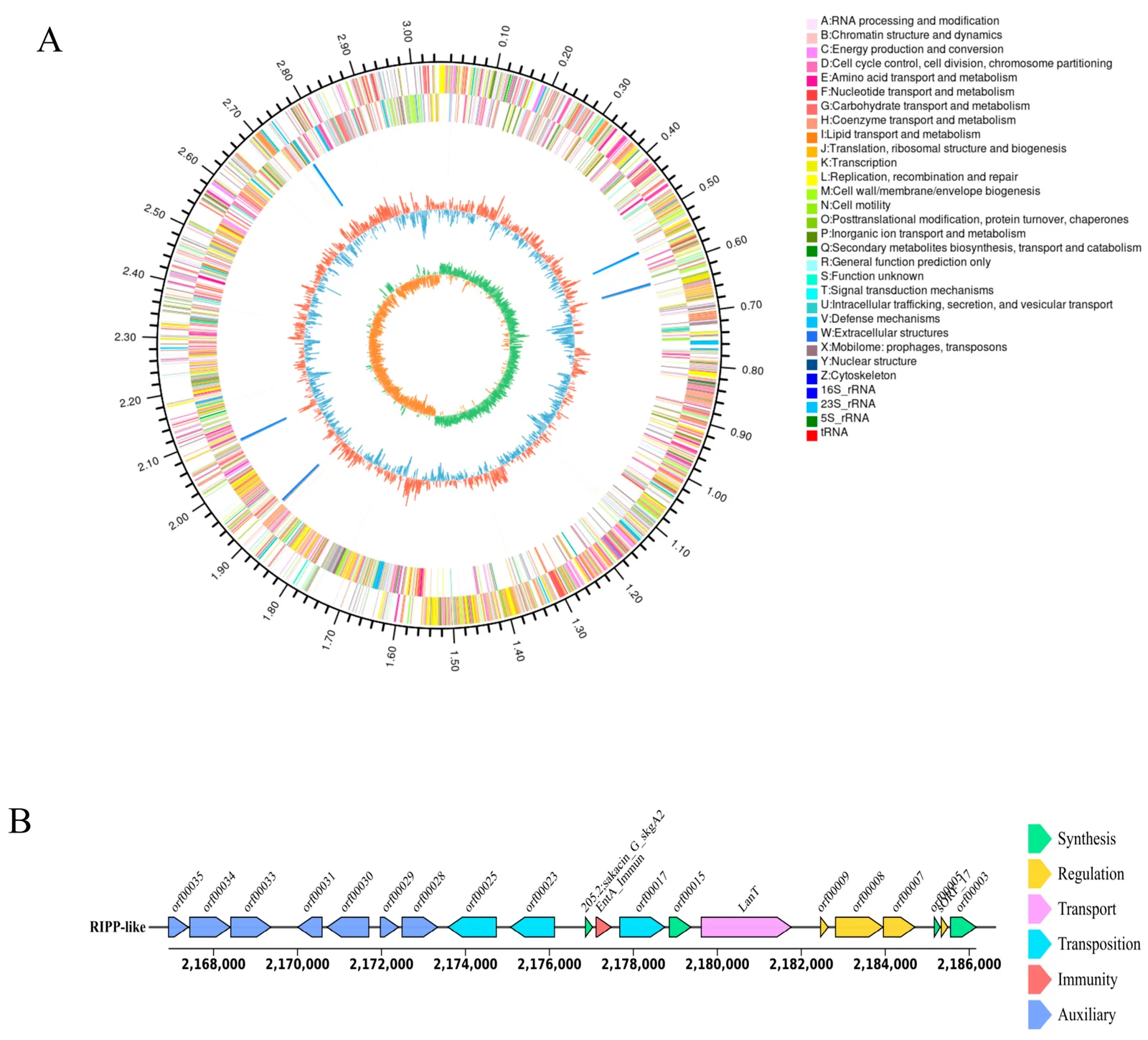
Genomic Analysis of *L*. *coryniformis* WYQ-2. (**A**) Genomic circle map of WYQ-2. (**B**) RiPP-like gene cluster of WYQ-2. To enhance clarity, only functional genes implicated in bacteriocin biosynthesis are presented in the figure, whereas non-functional or unannotated open reading frames have been omitted. Different functional modules are discriminated by color coding (refer to the figure legend for details), and the orientation of the arrows denotes the transcriptional direction.

The genome of strain WYQ-2 consists of one chromosome and four plasmids, totaling 2,702,355 bp with a GC content of 43.86%. It contains 3178 protein-coding genes, 64 tRNA genes, and 15 rRNA genes (5 copies each of 16S, 23S, and 5S rRNA). Sixty-four repetitive sequences (14,034 bp) were identified, accounting for 0.52% of the genome. Raw sequencing data are available under BioProject PRJNA1405893 and BioSample SAMN59686491. A circular genomic map constructed with Circos (Figure 4A) comprises six concentric rings: (1) the outermost scale ring; (2) forward-strand CDSs and (3) reverse-strand CDSs, color-coded by functional category; (4) rRNA and tRNA gene positions; (5) GC content, with red peaks indicating regions above the genome average and blue peaks regions below; and (6) GC skew [(G−C)/(G+C)], where positive skew suggests the replication origin and negative skew the replication terminus.

Whole-genome sequencing coupled with BAGEL4 analysis revealed a RiPP-like gene cluster within the genome of strain WYQ-2 (Figure 4B). The genetic architecture of this cluster is largely intact and encompasses multiple core functional modules indispensable for bacteriocin biosynthesis.

### 3.6. Molecular Weight Analysis

**Figure 5:**
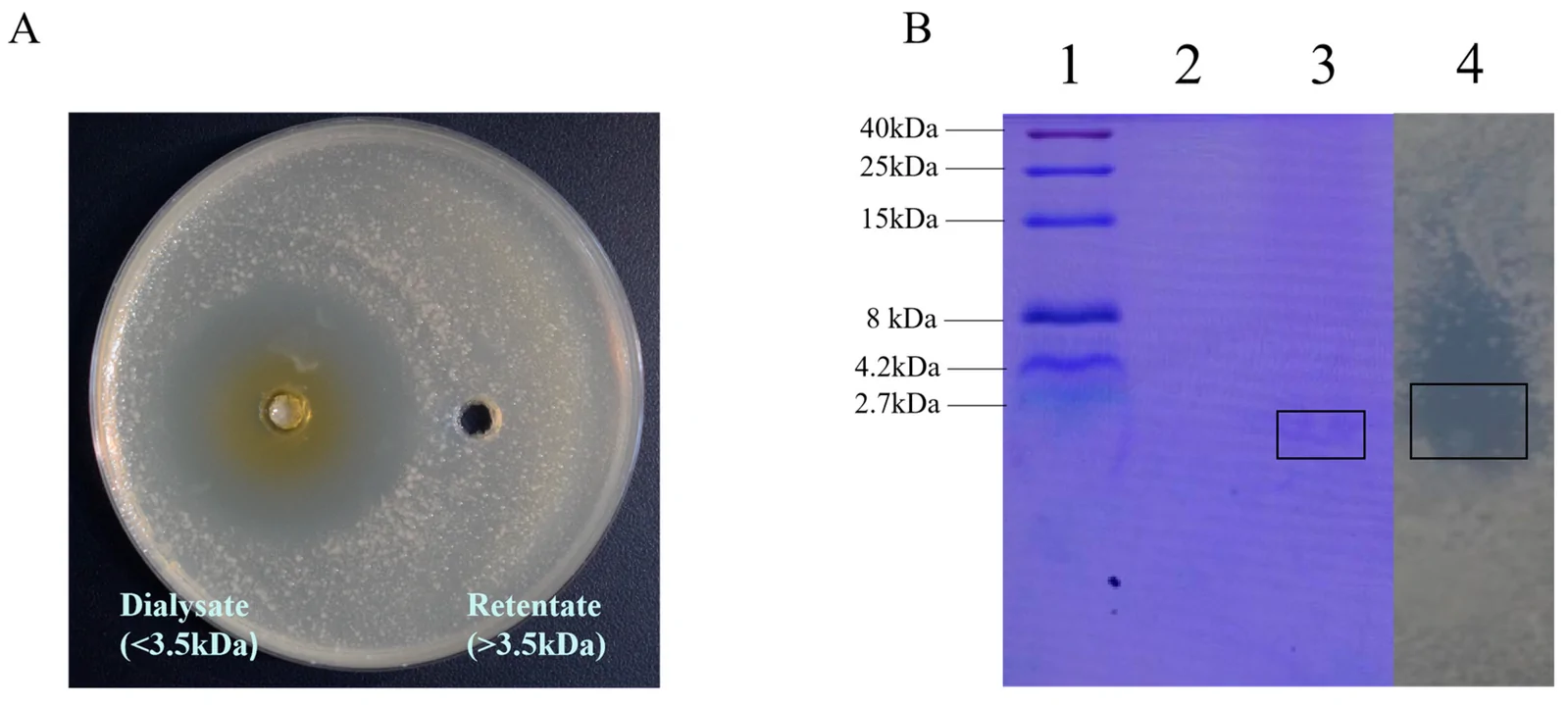
Molecular Weight Determination of BLIS WYQ-2cin from *L*. *coryniformis*. (**A**) Antibacterial activity of the dialysate (<3.5 kDa) versus the retentate (>3.5 kDa). (**B**) 1: Protein band on gel; (**B**) 2: PBS blank control; (**B**) 3: Low-molecular-weight protein marker; (**B**) 4: Antibacterial activity assay.

To determine the molecular weight range of BLIS WYQ-2cin, a combination of dialysis and electrophoretic approaches was employed. First, a crude extract of BLIS WYQ-2cin was fractionated using a dialysis membrane with a molecular weight cut-off of 3.5 kDa. After 24 h of dialysis, both the dialysate and the retentate were concentrated, and their antibacterial activities were compared. Agar diffusion assays (Figure 5A) revealed that the dialysate produced a distinct inhibition zone, whereas the retentate showed only minimal activity. Subsequently, the active fraction was analyzed by Tricine-SDS-PAGE. The electrophoretic profile displayed a single protein band migrating below the 2.7 kDa standard marker (Figure 5B). Further gel-overlay activity assay confirmed that the region corresponding to this band exhibited clear antibacterial activity. Together, these results demonstrate that BLIS WYQ-2cin is a small antimicrobial peptide with a molecular weight of less than 2.7 kDa.

### 3.7. Analysis of Intracellular ATP Quantification Results

**Figure 6:**
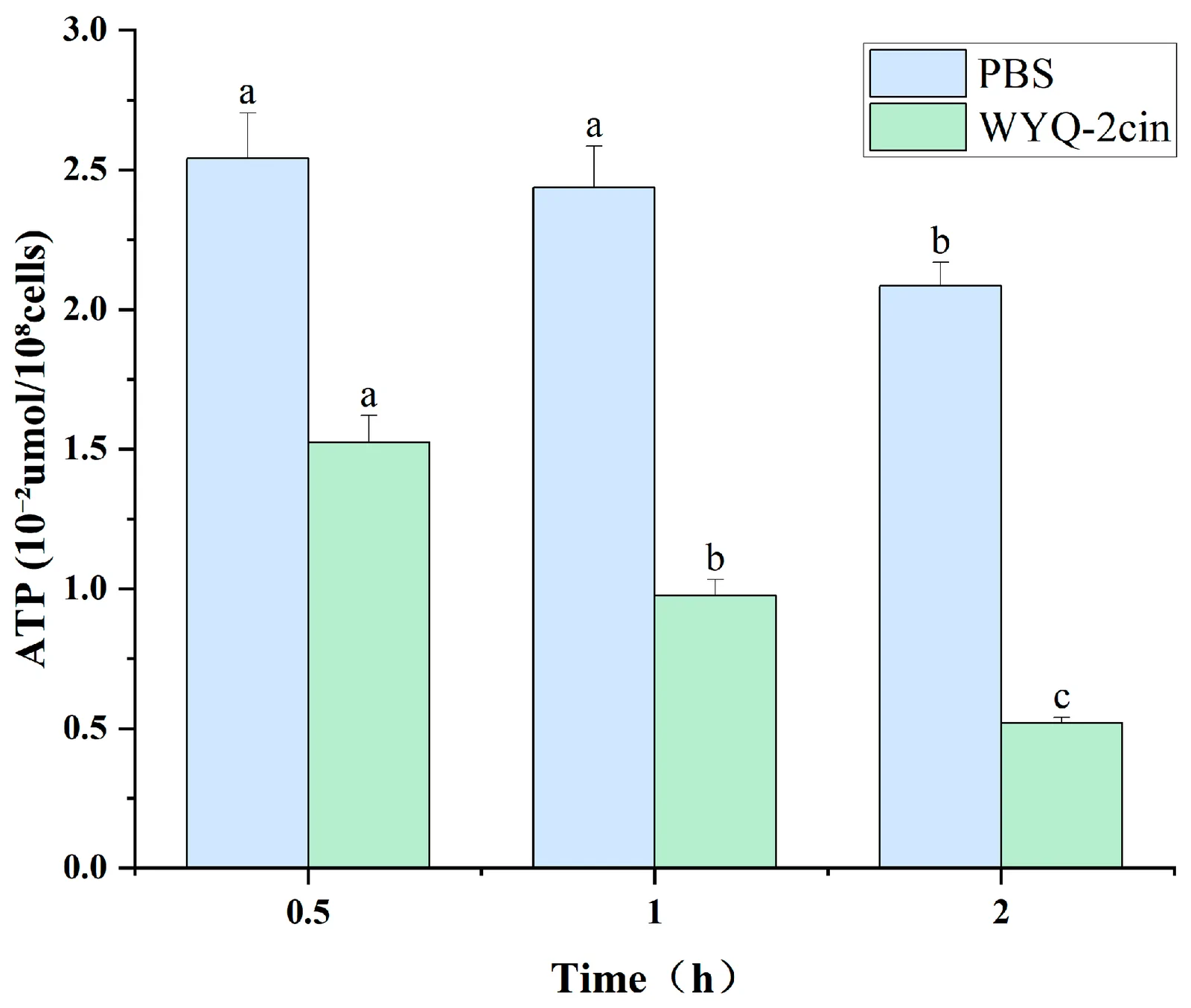
Effect of varying BLIS WYQ-2cin exposure durations on intracellular ATP levels in *E*. *coli* ATCC 25922 cells. Statistically significant differences (*p* < 0.05) among parameters are indicated by distinct letters.

The experimental results are depicted in Figure 6. Upon bacteriocin-like substance treatment, the intracellular ATP level diminished markedly, implying a potential compromise of the cellular integrity of *E*. *coli* ATCC 25922. At 0.5 h, the ATP concentration in the control group registered 0.25 μmol per 10^8^ cells and remained largely steady, with only a minor decline at 2 h. In contrast, the ATP concentration in the BLIS WYQ-2cin-treated group exhibited a conspicuous downward trajectory, confirming that the bacteriocin-like substance elaborated by WYQ-2 altered the membrane permeability of *E. coli* ATCC 25922 and consequently triggered ATP leakage.

### 3.8. Analysis of Live/Dead Cell Staining Results

**Figure 7:**
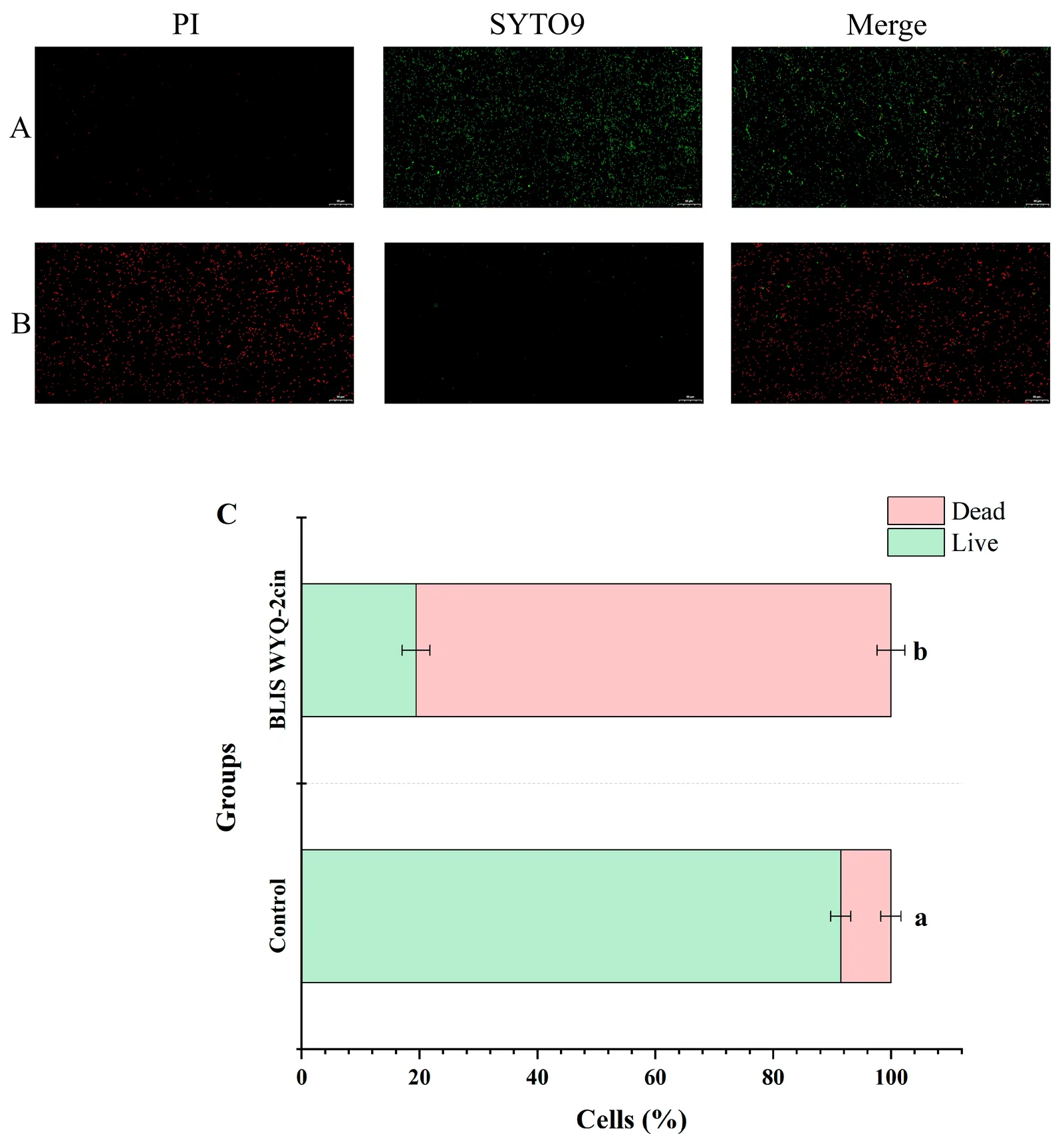
Fluorescence imaging after SYTO 9 and PI staining. (**A**) Control group without WYQ-2cin treatment. (**B**) Experimental group treated with WYQ-2cin. In each panel, the left image shows PI staining (red fluorescence), the middle image shows SYTO 9 staining (green fluorescence), and the right image displays the merged channels. (**C**) Fluorescence imaging-based quantification of cell viability. The stacked bar chart illustrates the relative proportions of live (green) and dead (red) cells. Statistically significant differences in the percentage of dead cells between the Control (left) and BLIS WYQ-2cin treated (right) groups are indicated by distinct letters (a, b) above the bars. Different letters indicate statistically significant differences (*p* < 0.05); the same letters indicate no significant difference (*p* ≥ 0.05).

The bactericidal effect of BLIS WYQ-2cin against *E*. *coli* ATCC 25922 was evaluated using a SYTO 9/propidium iodide (PI) dual-staining assay under fluorescence microscopy (Figure 7). In the untreated control (Figure 7A), the bacterial population showed predominantly green emission with only occasional red fluorescence, suggesting that the majority of cells retained membrane integrity and remained viable. Upon treatment with BLIS WYQ-2cin (Figure 7B), however, a pronounced elevation in red fluorescence was evident, revealing that the agent compromised membrane integrity and caused cellular damage. Furthermore, the quantitative analysis of the fluorescence imaging results was visualized as a stacked bar chart (Figure 7C). The findings revealed that the proportion of dead cells in the BLIS WYQ-2cin-treated group rose markedly relative to the control, with statistically significant differences, thereby corroborating the disruption of cell membrane integrity.

### 3.9. Analysis of Scanning Electron Microscope Results

**Figure 8:**
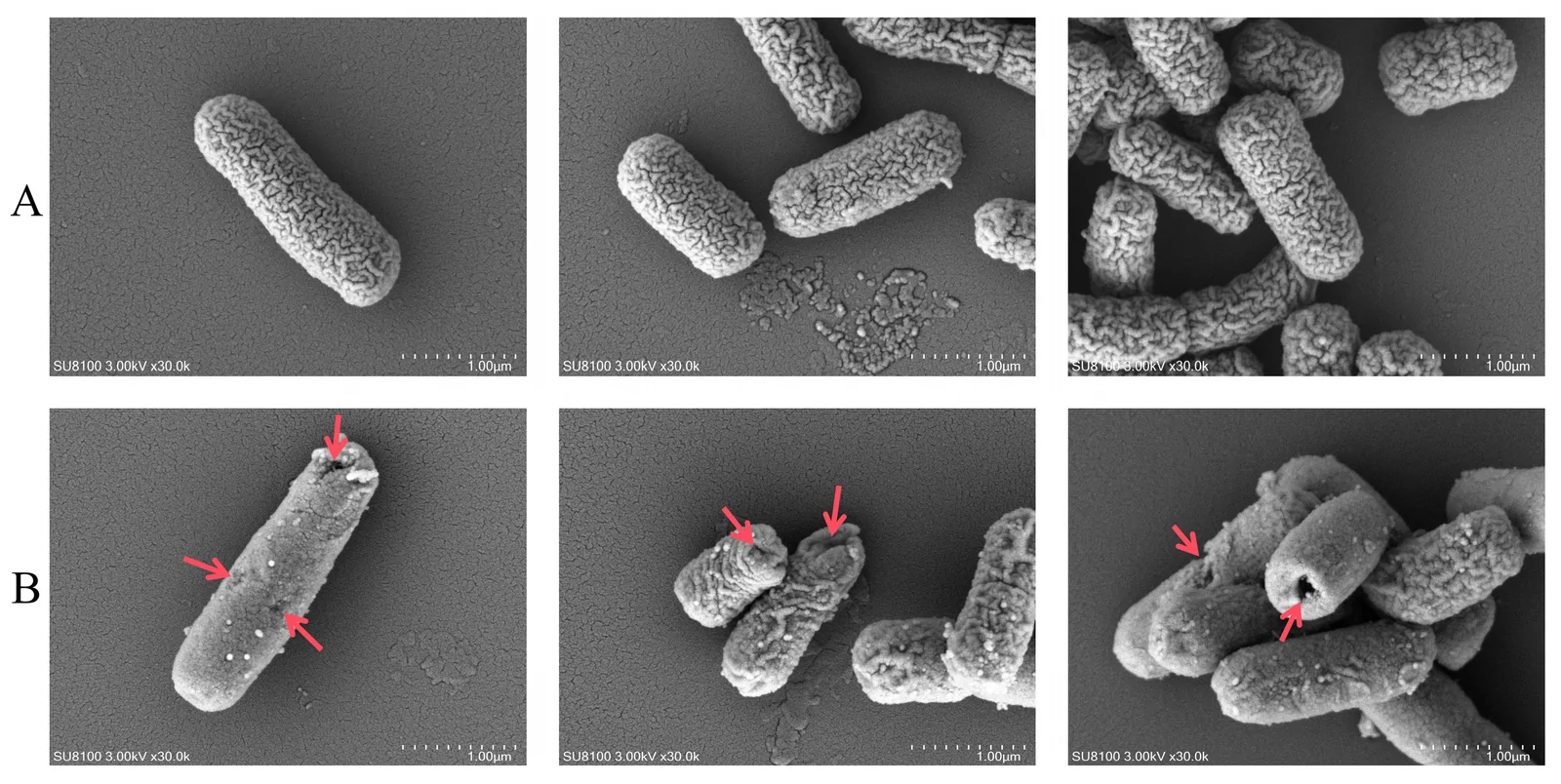
Analysis of SEM results. (**A**) *E*. *coli* ATCC 25922 treated with PBS for 2 h. (**B**) *E*. *coli* ATCC 25922 treated with BLIS WYQ-2cin for 2 h, showing surface depressions, ruptures, and collapse.

To investigate the bactericidal mechanism of BLIS WYQ-2cin, the ultrastructural morphology of treated bacterial cells was examined using scanning electron microscopy (SEM). Untreated control cells (Figure 8A) exhibited an intact, typical rod-shaped morphology. In contrast, after a 2 h exposure to an effective concentration of the antimicrobial agent, the treated bacterial cells (Figure 8B) showed distinct morphological alterations, including localized depressions, perforations, and a collapsed appearance. These structural damages indicate that the antimicrobial substance exerts its bactericidal effect by disrupting the integrity of the bacterial cell membrane.

## 4. Discussion

In this investigation, the physicochemical characteristics, genetic sequence, and antibacterial mechanism against porcine-origin diarrheagenic *E*. *coli* of the BLIS WYQ-2cin produced by *L*. *coryniformis* WYQ-2 isolated from pickled vegetables were examined. The findings demonstrated that the bacteriocin-like substance elaborated by this strain holds promise as an alternative to antibiotics, exhibiting prospective utility in managing piglet diarrhea attributable to *E. coli* infection.

The application potential of bacteriocins in livestock husbandry and feed is intimately tied to their physicochemical robustness. BLIS WYQ-2cin retained potent activity after exposure to 100 °C, exhibiting a thermostability profile characteristic of class II bacteriocins [22], which likely originates from its compact conformation, disulfide bonds, hydrophobic core, and other stabilizing intramolecular forces, enabling it to withstand high-temperature processing steps such as feed pelleting [23]. The bacteriocin-like substance remained active across a pH range of 3.0–9.0, a broad-spectrum trait that facilitates survival in the gastric acid environment and subsequent antibacterial action in the intestinal tract [24]. Furthermore, it was insensitive to 254 nm ultraviolet irradiation for 2.5 h, rendering it convenient to handle and store under non-light-protected conditions. Regarding protease stability, BLIS WYQ-2cin was highly susceptible to trypsin and papain, slightly sensitive to proteinase K, and resistant to pepsin and pronase. These differential sensitivities can be rationalized by considering both enzyme specificity and peptide structural features: trypsin exclusively hydrolyzes peptide bonds at the C-terminal side of lysine and arginine residues [25], papain cleaves a broad spectrum of peptide bonds involving basic and hydrophobic residues [26], and the N-terminal region of BLIS WYQ-2cin is enriched in lysine [27] and surface-exposed hydrophobic residues [28], thereby serving as an excellent substrate for these two enzymes; pepsin requires strongly acidic conditions (pH 2.0–3.0) for activation and may display suppressed activity under the near-neutral test environment, leading to reduced susceptibility [29]; pronase possesses limited hydrolytic capacity toward highly compact peptides [30]; proteinase K, although a broad-spectrum serine protease that preferentially targets exposed aliphatic and aromatic hydrophobic residues [31], encounters the compact folded architecture of BLIS WYQ-2cin that sequesters these hydrophobic sites within the molecular interior, likely impeding recognition and cleavage [32]. Collectively, the compact structure of BLIS WYQ-2cin endows it with simultaneous tolerance to processing and gastrointestinal transit as well as controlled enzymatic degradation in the gut, furnishing a favorable physicochemical foundation for its development as a feed additive. However, the proposed structural basis for these resistance patterns requires further verification through high-resolution structural analysis.

The molecular mass analysis demonstrated that BLIS WYQ-2cin is a low-molecular-weight bacteriocin (<2.7 kDa), considerably smaller than the typical mass range reported for most *L. coryniformis* bacteriocins (3–10 kDa), such as *curvacin A* (4.2–4.5 kDa) and *sakacin P* (4.5 kDa) [33]. Although a few exceptionally small bacteriocins have been documented in other lactic acid bacteria—for instance, the antimicrobial peptide (1.5 kDa) produced by *Bacillus licheniformis* MKU3 [34] and plantaricin UG1 (1.8 kDa) from *Lactiplantibacillus plantarum* UG1 [35]—such ultra-low-molecular-mass bacteriocins remain rare among LAB. This diminutive molecular size facilitates penetration of the outer membrane barrier of Gram-negative bacteria and subsequent interaction with the cytoplasmic membrane [36], which may represent the structural foundation accounting for its potent antibacterial activity against Gram-negative organisms.

The antimicrobial spectrum assessment revealed that BLIS WYQ-2cin displayed broad inhibitory activity against both Gram-negative and Gram-positive bacteria, with its antibacterial potency toward Gram-negative strains generally surpassing that observed against Gram-positive ones. Notably, the most pronounced inhibition was recorded against *E*. *coli* ATCC 25922. Consequently, the potent antagonistic activity of this bacteriocin-like substance toward Gram-negative bacteria merits particular attention—given that bacteriocins originating from lactic acid bacteria are typically more effective against Gram-positive organisms owing to the permeability barrier imposed by the outer membrane of Gram-negative prokaryotes [37]; however, certain Class II bacteriocins can breach the outer membrane of Gram-negative bacteria through electrostatic interactions and membrane disruption mechanisms [38]. The marked inhibitory effect of BLIS WYQ-2cin against *E. coli* strongly suggests that it likely belongs to the Class II bacteriocins, a deduction that aligns well with its documented molecular mass and stability characteristics. However, the analysis of the antibacterial mechanism of BLIS WYQ-2cin in this study was primarily based on the model strain *E. coli* ATCC 25922. Although antimicrobial spectrum experiments have confirmed its efficacy against multiple clinical isolates of swine-derived *E. coli*, it remains to be further verified whether subtle differences in cell membrane structure among different strains affect their susceptibility.

Following initial characterization of the antimicrobial substance produced by strain WYQ-2, whole-genome sequencing was undertaken to identify the cognate gene cluster. Subsequent analysis of the sequencing data revealed a RiPP-like gene cluster. Detailed investigation of this cluster yielded the following findings: it is situated in chromosomal region 3, with a total length of 14,903 nt (positions 2,167,188–2,182,090 nt), and encodes a comprehensive bacteriocin biosynthetic machinery encompassing functional modules for precursor peptide processing and maturation, quorum-sensing-dependent regulation, transmembrane export, self-immunity protection, and genetic plasticity [39,40]. Within the synthetic module, *orf00003* encodes a membrane-anchored CAAX protease (CPBP superfamily) responsible for excising the N-terminal leader region of the precursor peptide to release the active core, thereby constituting a critical rate-limiting step for mature peptide bioactivity [41]. The *orf00005* and *sORF_11* (*sakacin_G_skgA2*) jointly serve as precursor peptide-encoding genes, and their products both harbor a highly conserved double-glycine cleavage signal [39]; notably, *orf00005* simultaneously possesses structural domain features characteristic of both lantibiotics and class II bacteriocins, implying a potential multi-precursor cooperative processing mode within this gene cluster. The *orf00015* is annotated as a homolog of PedC, a biosynthetic protein involved in pediocin PA-1 production, and may participate in bacteriocin maturation [42]. In the regulatory module, *orf00007* (encoding the response regulator SppR) and *orf00008* (encoding a histidine kinase sensor) constitute a two-component phosphorelay system that functionally mimics the Agr quorum-sensing mechanism [43]: the kinase perceives an extracellular quorum signal, undergoes trans-autophosphorylation, and subsequently transfers the phosphoryl group to the cognate regulator, thereby amplifying the transcriptional activation signal in a positive-feedback manner and enabling dynamic gene expression in response to bacterial population density [44,45]. The *orf00009* and *sORF_17* also participate in this regulatory network, collectively coordinating transcription of the gene cluster. Regarding transport, *orf00012* (*LanT*) encodes an ABC-type transporter harboring an ATPase domain (PF00005) and a transmembrane domain (PF03412), which mediates energy-dependent translocation of the mature peptide across the membrane [46]. The immunity module is provided by *orf00019* (*EntA_Immun*), which specifies a membrane-integral protein (PF08951) capable of specifically neutralizing the membrane-perforating activity of the cognate bacteriocin, thereby protecting the producer strain from self-killing [47,48]. In the mobility module, *orf00017* and *orf00025* encode transposases of the IS1161 and Tn1545 families, respectively, and *orf00023* encodes the DNA relaxase *NicK*; together, these three elements confer genetic transfer potential to the gene cluster. Additionally, *orf00028*–*orf00035* consecutively encode response proteins, Clp proteases, gluconeogenesis factors, and other auxiliary proteins that supply physiological support—including energy provision and protein homeostasis—for bacteriocin synthesis and secretion [49,50]. Collectively, this gene cluster integrates a complete functional chain encompassing regulation, transport, and immunity, and represents a prototypical class II bacteriocin biosynthetic system [51]. Previous research has demonstrated that class II bacteriocins are small, heat-stable antimicrobial peptides—a feature fully congruent with the observed thermostability and molecular mass characteristics of BLIS WYQ-2cin [39]. Given the presence of multiple precursor peptide-encoding genes carrying double-glycine-type leader sequences within this cluster (e.g., *sORF_11*, *orf00005*, and *orf00009*), it can be deduced that this gene cluster encodes a class II bacteriocin whose mature peptide is a low-molecular-mass antimicrobial peptide.

Regarding growth kinetics, the WYQ-2 strain entered the stationary phase after 20 h of cultivation, at which point the pH of the fermentation broth had stabilized at approximately 2.5, following a marked decline that began after the initial 8 h. As for antibacterial activity, an inhibition zone was first observed at 8 h (9 mm in diameter), gradually intensified thereafter, and attained a maximum of 18.9 mm at 26 h, a time point that precisely coincided with the stationary growth stage of the strain. These dynamics indicate that bacteriocin accumulation occurred in a growth-associated manner, which is consistent with the pattern typically exhibited by antimicrobial substance-producing lactic acid bacteria [52].

The antibacterial mechanism of BLIS WYQ-2cin against porcine diarrheagenic *E*. *coli* ATCC 25922 was substantiated at multiple levels. Scanning electron microscopy revealed that after 2 h of bacteriocin-like substance exposure, the target cell surfaces exhibited conspicuous indentations, pore formation, and overall collapse, indicating disruption of cellular morphological integrity. Furthermore, SYTO-9/PI dual staining displayed a pronounced increase in red fluorescence accompanied by diminished green fluorescence, demonstrating that the membrane integrity of a large proportion of bacterial cells was compromised. Subsequent quantification of intracellular ATP content showed that the ATP concentration in the treated group declined continuously over time, with a marked reduction between 0.5 h and 2 h, suggesting that altered membrane permeability resulted in ATP leakage. Coupled with the time-kill kinetics showing that OD_600_ values remained stable over 24 h without any discernible resurgence, it can be concluded that BLIS WYQ-2cin exerted a bactericidal effect. Collectively, these findings—including morphological aberrations, loss of membrane barrier function, leakage of intracellular small molecules, and irreversible growth inhibition—converge to indicate that BLIS WYQ-2cin effectively eradicates the target bacterium by disrupting the integrity of the *E. coli* cytoplasmic membrane and eliciting the efflux of intracellular contents. This mode of action aligns with the membrane-perforating mechanism characteristic of typical class II bacteriocins [53] and provides direct experimental evidence for its potential application in the control of porcine bacterial diarrhea.

Despite the experiments conducted, this study still has certain limitations. First, the “purification” of BLIS WYQ-2cin actually involved only concentration and dialysis rather than rigorous chromatographic purification; therefore, the active fraction obtained should be regarded as an “enriched preparation” rather than a highly purified single compound. Owing to this constraint, the antibacterial activity evaluation in this study mainly relied on the inhibition zone diameter as a screening indicator, and MIC, MBC, and dose–response relationships were not determined; these quantitative assessments and structural characterization should be systematically conducted once chromatographically purified samples become available. Second, the analysis of the antibacterial mechanism in this study primarily depended on intracellular ATP levels, live/dead cell staining, and SEM morphological observations. Although the results revealed that ATP levels began to decline as early as 0.5 h, whereas evident cell surface damage did not appear until 2 h later, suggesting that altered membrane permeability is an early event rather than secondary structural damage occurring in the late stage of cell death, the aforementioned findings still constitute indirect evidence and cannot completely rule out the possibility that membrane damage is merely a concomitant phenomenon in the terminal phase of apoptosis or necrosis. Future investigations are warranted to employ membrane potential fluorescent probes, quantification of intracellular nucleic acid/protein leakage, and K^+^ efflux measurements to further confirm whether membrane disruption represents the specific bactericidal mechanism of BLIS WYQ-2cin and to evaluate whether it can function stably in complex in vivo environments.

The physiological environment encountered by BLIS WYQ-2cin within the living intestine is considerably more intricate than that in vitro, and factors such as digestive enzyme degradation, pH fluctuations, the host immune barrier, and competitive exclusion by the resident microbiota may collectively attenuate its antibacterial activity and targeting efficiency. Although in vitro data have convincingly demonstrated its potent direct bactericidal capacity, whether it can maintain an effective concentration and duration of action in the in vivo environment is the decisive factor for achieving practical prevention and control outcomes. Therefore, subsequent studies should utilize animal models to validate its genuine therapeutic efficacy and to enhance its survivability and functional stability under the harsh intestinal environment.

## 5. Conclusions

In conclusion, the present study comprehensively characterized the in vitro physicochemical properties and antimicrobial potential of the BLIS WYQ-2cin produced by *L. coryniformis* WYQ-2. The strain exhibited excellent tolerance to acidic and alkaline conditions. Of particular importance, a class II bacteriocin produced by WYQ-2 was identified and shown to exert potent inhibitory activity against *E*. *coli*. This bacteriocin-like substance maintained stable antimicrobial activity across a broad temperature and pH ranges, demonstrating favorable physicochemical properties. Preliminary investigations suggest that its antibacterial mechanism may involve disrupting the integrity of the pathogen’s cell membrane. Based on its significant in vitro antimicrobial efficacy and stability, this bacteriocin-like substance shows promise as a potential antibiotic alternative, warranting further in vivo evaluation in animal models for the control of porcine bacterial diarrhea. These in vitro findings provide a solid theoretical and experimental foundation for further evaluation of the active substance produced by strain WYQ-2 in animals and also explore new potential pathways for its practical application against intestinal pathogens in the livestock industry.

## Data Availability

The original contributions presented in this study are included in the article and Supplementary Materials. Further inquiries can be directed to the corresponding authors.

## Supplementary Materials

The following supporting information can be downloaded at: https://www.mdpi.com/article/10.3390/vetsci13090909/s1, Figure S1: Original full-length gel imagefor Figure 5B, 1, 2, 3.
