# Supplementary material for: Characterization of a Bacteriocin-like Substance from Loigolactobacillus coryniformis WYQ-2 and Its Antibacterial Activity Against Escherichia coli
Source: Vet Sci. 2026 Sep 4;13(9):909. doi: 10.3390/vetsci13090909 (PMC13612013; doi:10.3390/vetsci13090909)
Supplement: Supplementary file 1 [file vetsci-13-00909-s001.zip › vetsci-4472319-supplementary Figure S1.pdf]

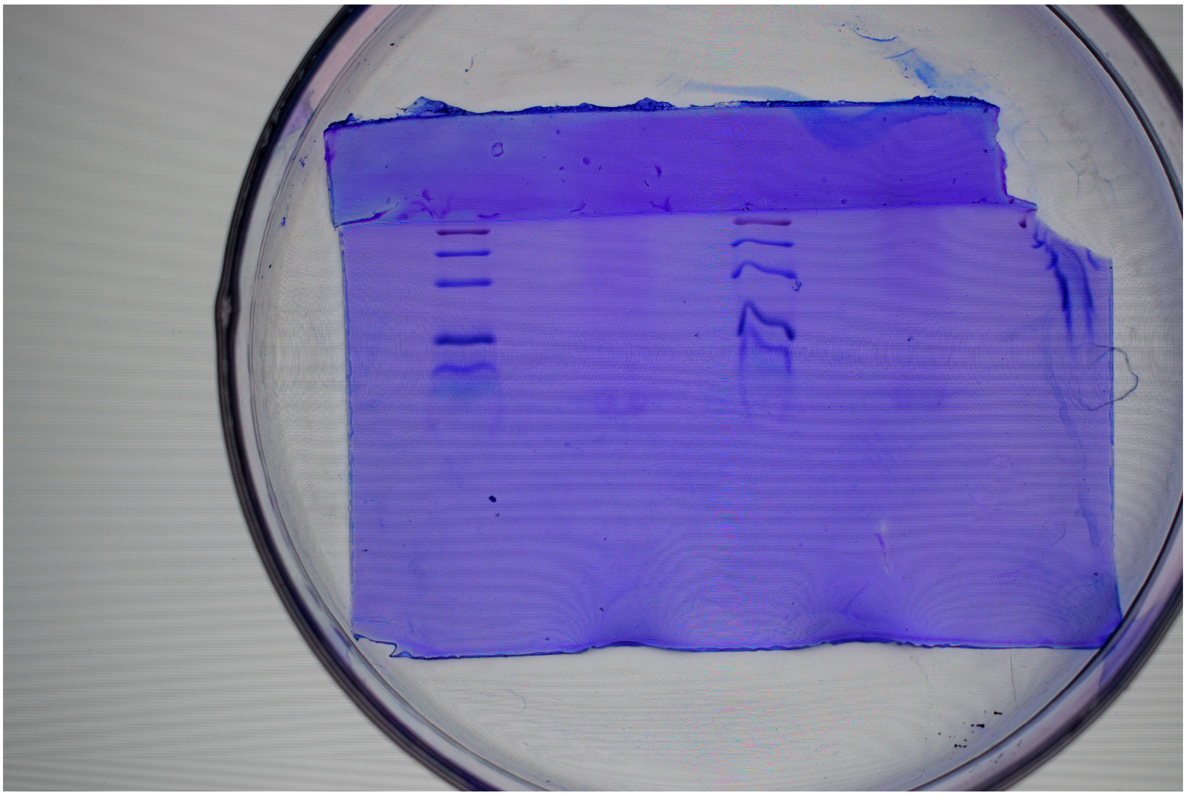

Figure S1. Original full-length gel imagefor Figure 5B, 1, 2, 3.
